# Acceptability, appropriateness, sustainment, and implementation cost of a multicomponent strategy to increase HPV vaccination in pediatric primary care: secondary results of a cluster-randomized trial

**DOI:** 10.1186/s43058-026-01068-w

**Published:** 2026-08-06

**Authors:** Ruoyun Wang, Sherry Dodd, Shannon Rook, Sharon Graham, Abigail R. Barker, Katie Plax, Michelle I. Silver, Allison A. King

**Affiliations:** 1 Department of Pediatrics, Washington University School of Medicine in St. Louis, MSC 8505-45-01, 660 S. Euclid Ave, St. Louis, MO 63110 USA; 2 Bursky School of Public Health, Washington University, St. Louis, MO USA; 3 Division of Infectious Disease, Department of Pediatrics, Nationwide Children’s Hospital, Columbus, OH USA; 4 Division of Public Health Sciences, Department of Surgery, Washington University School of Medicine, St. Louis, MO USA

**Keywords:** Implementation outcomes, Sustainment, Cost analysis, Practice facilitation, HPV vaccination

## Abstract

**Background:**

Implementation outcomes and cost are as essential as clinical effectiveness for evidence-based practice adoption, yet few HPV vaccination improvement studies have assessed both using validated tools. Moreover, sustainment remains understudied in randomized controlled trials beyond the active implementation period. This gap limits the ability of health systems to make informed decisions about adoption and scale-up of the implementation strategy.

**Objective:**

To assess implementation outcomes, including acceptability, appropriateness, sustainment, and estimate implementation costs from two stakeholder perspectives for a multicomponent strategy designed to improve HPV vaccination in pediatric primary care practices.

**Methods:**

We conducted a secondary analysis of a 24-month cluster randomized controlled trial involving 20 pediatric practices. Implementation outcomes were assessed among providers using validated tools post-implementation. Because implementation outcomes were assessed following exposure and at different time points across study groups, analyses reflect provider perceptions of the strategy rather than causal comparisons. Implementation costs for the 10 intervention practices were evaluated from two perspectives: practice facilitators and practice personnel. Costs were calculated using personnel time multiplied by national wage rates from the 2023 U.S. Bureau of Labor Statistics. We used a micro-costing approach to estimate implementation resource use. Cost-effectiveness was assessed as cost per percentage-point improvement per practice and cost per additional adolescent vaccinated.

**Results:**

Among 43 post-implementation survey responses (60% response rate), mean scores of acceptability (4.33 of 5) and appropriateness were high (4.37 of 5), with positive sustainment (3.95 of 5). Mean implementation cost was $10,021 per practice, requiring 93 h of facilitator time and 44 h of practice personnel time per practice over 24 months. Cost per additional adolescent completing the HPV vaccination series was $527 (facilitator: $238, practice: $289). These findings suggest favorable implementation outcomes alongside transparent, dual-perspective resource estimates.

**Conclusions:**

The multicomponent strategy demonstrated high acceptability and appropriateness among providers, minimal disruption to clinical operations, and maintained positive sustainment ratings 12 months post-implementation. By reporting provider perceived implementation outcomes with detailed cost data from both implementer and practice perspectives, this study provides actionable evidence to inform feasibility and scalability of HPV vaccination implementation strategies in pediatric primary care.

**Trial registration:**

ClinicalTrials.gov NCT04180462.

**Supplementary Information:**

The online version contains supplementary material available at https://doi.org/10.1186/s43058-026-01068-w.

Contributions to the literature

- Few randomized trials evaluate implementation outcomes, including acceptability, appropriateness, and sustainment after active implementation delivery concluded, alongside detailed implementation cost estimates from both implementer and recipient perspectives for a multicomponent implementation strategy.
- This study links implementation outcomes with micro-costing in a pragmatic trial, advancing understanding of how perceived implementation strategy fit aligns with real-world resource requirements for sustained implementation.
- These findings provide actionable evidence to inform scalability and adoption of facilitation-based implementation strategies in pediatric primary care.

## Background

Despite strong evidence supporting the effectiveness of HPV vaccination in preventing HPV-related cancers, uptake remains suboptimal in pediatric primary care, highlighting a persistent gap between evidence and routine practice [1, 2]. Numerous implementation strategies have been developed to close this evidence-to-practice gap and improve HPV vaccination delivery [3]. While demonstrating their effectiveness is the first critical step, it alone is insufficient for ensuring widespread adoption [4]. Implementation strategies must also be acceptable, appropriate, and feasible to sustain within healthcare settings. Without attention to these factors, even effective strategies may fail to achieve population-level impact.

To address the suboptimal HPV vaccination uptake in primary care pediatric practices [1], we previously conducted a cluster randomized controlled trial (RCT) aimed at improving adherence to the Centers for Disease Control and Prevention (CDC) guidelines, which recommend completing HPV vaccination by 13 years of age [5]. The strategy incorporated components aligned with the Expert Recommendations for Implementing Change (ERIC) taxonomy [6], such as practice facilitation and educational outreach, to address barriers to HPV vaccination over 24 months, including workflow inefficiencies, limited performance feedback, provider communication challenges, and integration supports into routine care [7]. The primary analysis showed that effectiveness of the strategy progressively increased over time, with the largest effect magnitude observed during the 12-month sustainment follow-up. To understand what drove this pattern and whether the strategy is feasible for broader adoption, evaluation of implementation outcomes is needed.

Proctor’s Implementation Outcomes Framework identifies acceptability, appropriateness, sustainment, and cost as critical preconditions for successful adoption, providing insights into how and why evidence-based practices succeed [8]. Despite their importance, these outcomes, particularly sustainment, remain understudied in randomized trials of HPV vaccination strategies [3, 9]. 

Even when implementation outcomes are favorable, a persistent barrier to scaling practice-based strategies is the lack of transparent cost information [10, 11]. Decision-makers require implementation cost estimates, including personnel time and resource requirements, to assess feasibility and plan for scale-up. However, these costs remain underreported in practice due to variation in costing methods and unclear boundaries around what constitutes an implementation expense [11]. Practice facilitation strategy, while effective at producing lasting change, require sustained resource investment [12], making cost transparency particularly critical for widespread adoption [13]. Yet, detailed cost analyses of multicomponent, facilitation-based strategy remains scarce in the HPV vaccination literature. Published cost data have focused on less intensive strategies, with limited evidence on the resource requirements of sustained practice facilitation models [14–17]. In addition, few studies estimate costs from both the implementer (e.g., facilitation program) and recipient (e.g., clinical practice) perspectives, limiting the ability of health systems to plan for adoption and scale-up.

Therefore, we performed this secondary analysis to assess the implementation outcomes of our multicomponent strategy with two objectives: (1) to assess acceptability, appropriateness, and sustainment among providers who received the strategy, and (2) to provide detailed cost estimates of the 24-month implementation strategy to inform scaling decisions from both the practice facilitator and pediatric practice perspectives. By pairing provider-reported implementation outcomes (acceptability, appropriateness, and sustainment) with detailed cost data from a pragmatic RCT, this study addresses a critical gap between implementation effectiveness and real-world scalability, offering insight into how perceived fit aligns with real-world resource investment required for sustainment and scale-up of HPV vaccination strategies in pediatric primary care.

## Methods

### Strategy overview

This secondary analysis examined implementation outcomes from a cluster RCT that was conducted in the St. Louis, Missouri metropolitan area from 2020 to 2022, comprising a 24-month active implementation followed by a 12-month sustainment period (2022–2023). Details of the trial can be found elsewhere [5]. Briefly, the study included 20 pediatric primary care practices (10 intervention, 10 control), with a total of 86 providers. We examined a multicomponent strategy to improve HPV vaccination completion by 13 years of age. Aligned with the ERIC taxonomy, the strategies included: (1) practice facilitation by trained external facilitators, (2) audit and feedback of vaccination rates, (3) HPV vaccination education, and (4) an announcement-style communication strategy. Implementation strategies were conducted either in-person or via Zoom video conferencing, adapted for COVID-19 safety protocols. After the conclusion of the intervention group’s sustainment follow-up (2022–2023), we offered the waitlist control group the same multicomponent strategy for 12 months, and 9 of the 10 control practices received it between 2023 and 2024. In May 2025, a single post-implementation survey was administered to both groups. The study was reviewed and approved by the Institutional Review Board of Washington University in St. Louis.

**Fig. 1:**
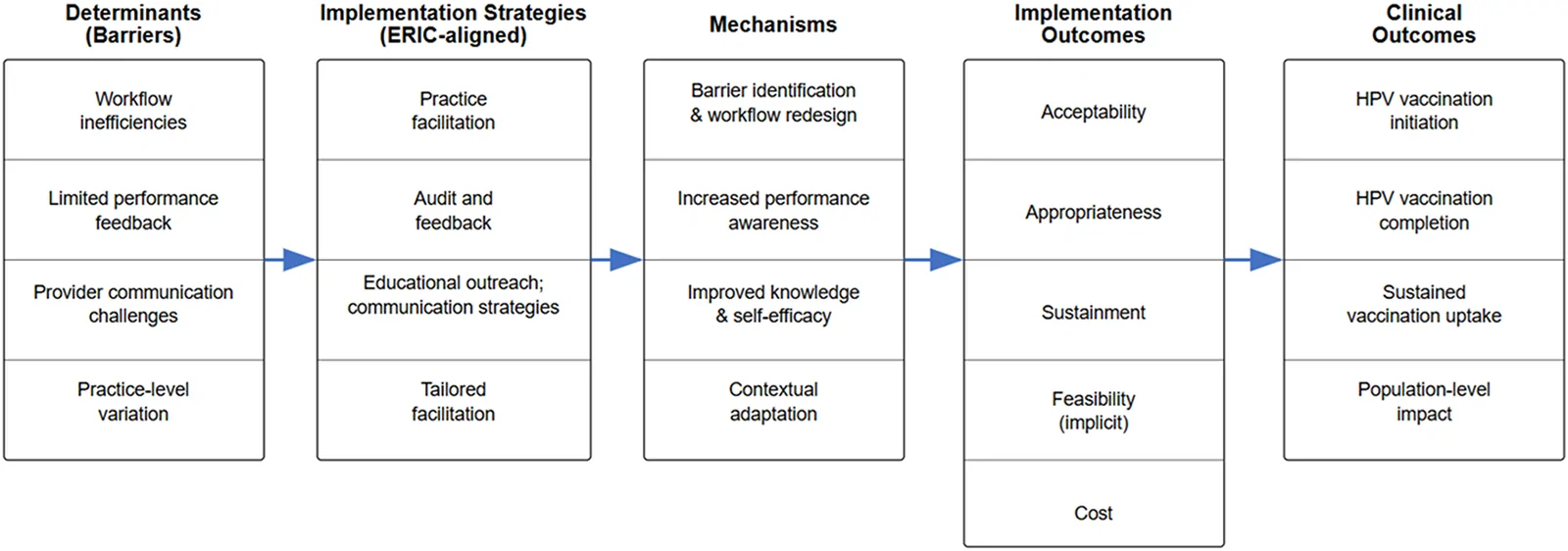
Implementation Research Logic Model for the HPV vaccination improvement strategy. Implementation strategies are classified using the Expert Recommendations for Implementing Change (ERIC) taxonomy

We conceptualized this study using the Implementation Research Logic Model (IRLM) [18], which specifies relationships among implementation determinants, strategies, mechanisms, and outcomes (Fig. 1). Specifically, practice facilitation and audit and feedback were expected to address organizational and workflow-related determinants, while education and communication strategies targeted provider knowledge, attitudes, and recommendation practices. We hypothesized that these strategies would operate through mechanisms including barrier identification and resolution, increased performance awareness, and improved provider confidence, leading to enhanced acceptability and appropriateness of the strategies. These proximal implementation outcomes were expected to support sustainment of practice changes and ultimately contribute to improved HPV vaccination rates. Consistent with IRLM guidance, this model was used not only to guide implementation strategy design but also to inform interpretation of implementation outcomes and cost findings by linking observed results to hypothesized mechanisms and resource inputs.

### Implementation outcomes assessment

We assessed implementation outcomes across multiple levels. Acceptability, appropriateness, and sustainment were measured at the provider level, and implementation cost at the practice level. Cost-effectiveness estimates were then derived by relating implementation cost to clinical effectiveness, reported from both practice level and patient level. This reflects the multi-level nature of our multicomponent strategy.

### Provider-reported implementation outcomes

In May 2025, 72 providers at the 17 of 20 practices (3 practices were lost to follow-up) were invited to complete a post-implementation survey assessing acceptability, appropriateness, and sustainment, administered via Research Electronic Data Capture (REDCap) [19, 20]. Survey responses were pooled to evaluate overall provider perceptions toward the implementation strategy. Because implementation outcomes were assessed following exposure to the strategy and timing differed between groups, analyses reflect provider perceptions of the strategy rather than causal comparisons between trial groups.

The survey assessed three key implementation outcomes using validated instruments. Acceptability and appropriateness were adopted from Acceptability of Intervention Measure (AIM) and the Intervention Appropriateness Measure (IAM) [21]. Each measure comprises four questions rated on a 5-point Likert scale (1 = completely disagree, 5 = completely agree). Sustainment was assessed using three questions from Provider REport of Sustainment Scale (PRESS) [22] rated on a 5-point Likert scaled on agreement (1 = not at all, 5 = to a very great extent).

Implementation outcomes were analyzed using descriptive statistics. Internal consistency was assessed using Cronbach’s alpha. Given the descriptive aim of this analysis, no between-group hypothesis testing was performed. Analyses were conducted using R 4.5.0 (R Group for Statistical Computing).

### Cost and cost-effectiveness

#### Implementation costs

We evaluated implementation costs from two major stakeholders (perspectives) that followed the recommended evidence-based implementation cost evaluation [23]. The practice facilitator perspective reflects the costs required to deliver the implementation strategy from the implementer’s viewpoint. It captured the external facilitators’ time spent delivering the strategy across all components, disaggregated by specific activity: quality improvement meeting, including facilitator-led meetings and Plan-Do-Study-Act cycles; interviews with providers to assess practice needs and implementation progress; training and coaching, including educational vaccine in-services for staff and providers; and data collection, including chart audits and data extraction for vaccination rate feedback.

The pediatric practice perspective represents the opportunity costs of practice personnel time devoted primarily to these same strategy activities, such as meetings, interviews, and training, from the recipient’s viewpoint. Across the two perspectives, facilitator time was organized by activity to reflect how the strategy was delivered, whereas practice personnel time was organized by staff role to reflect who participated. The combined perspective represents the total implementation cost, allowing more accurate estimation of real-world resource requirements for scale-up.

Implementation costs reflected only the resources required to deliver the multicomponent strategy. We excluded the cost of the HPV vaccine, its administration, routine clinical care, as well as pertaining to the research itself. Costs were systematically tracked by 2 practice facilitators for all 10 practices in the intervention group throughout the 24-month implementation period. We used a micro-costing approach to estimate implementation costs, capturing detailed personnel time and resource utilization associated with each strategy component. Personnel costs were estimated by multiplying hours spent by position-specific mean hourly wages from the U.S. Bureau of Labor Statistics (2023), matched to occupational categories, including primary care physicians (PCPs), nurse practitioners, registered nurses, medical assistants, practice managers, and practice facilitators. To reflect the total compensation, hourly wages were then adjusted to include benefits by multiplying by 1.416 [24]. Transportation cost was valued at the Internal Revenue Service (IRS) business mileage reimbursement rate of $0.655/mile [25]. 

#### Cost-effectiveness

Cost-effectiveness was assessed at both practice and patient level for HPV vaccination initiation and series completion, using effectiveness measured at the sustainment period (12 months after active strategy delivery concluded). As control practices (usual care) were not exposed to the multicomponent strategy until after this period, they incurred no implementation cost; their implementation cost was therefore assumed to be zero. The mean implementation cost per practice represents the incremental cost of the multicomponent strategy relative to usual care.

At the practice level, cost per percentage-point improvement was calculated as mean implementation cost per practice divided by the percentage-point difference between intervention and control groups at sustainment period. Based on the baseline-adjusted model from the prior RCT, the between-group difference was 6.2 percentage points for HPV vaccination initiation and 8.6 percentage points for series completion.

At the patient level, cost per additional adolescent vaccinated was calculated as total implementation cost across 10 practices divided by the estimated number of additional vaccinations (percentage-point difference multiplied by 2,211 eligible intervention group adolescents at the sustainment period).

Both metrics were reported from the practice facilitator, pediatric practice, and combined perspectives. The numerator reflects the cumulative implementation cost incurred across the full 24-month active delivery period, whereas the denominator reflects the between-group effectiveness difference measured at the sustainment timepoint (12 months after active strategy delivery concluded). These quantities therefore span different time horizons, and the resulting ratios should be read as the cost of achieving a given outcome rather than as time-matched incremental cost-effectiveness ratios. These estimates are intended to inform resource planning for adoption and were not evaluated against an established cost-effectiveness benchmark.

## Results

### Provider-reported implementation outcomes

A total of 43 providers from 15 practices (60% response rate) completed the post-implementation survey. Respondents were primarily physicians (79%), female (77%), White (86%), with a median of 21 years in practice. Most practices were metropolitan (95%) and served patients with a median of 20% Medicaid coverage (Table 1).

**Table 1:** Practice and provider characteristics from post-implementation survey

Characteristics	
**Practice**, ***n***	**15**
**Location**, ***n* (%)**	
Metropolitan	14 (95)
Rural	1 (5)
**Medicaid**,** median (IQR)**,** %**	20.0 (5.0-37.5)
**Provider**,** n**	**43**
**Profession**,** n (%)**	
MD/DO	34 (79)
Nurse practitioner	9 (21)
**Sex**,** n (%)**	
Female	33 (77)
Male	10 (23)
**Race**,** n (%)**	
White	37 (86)
Asian	3 (7)
Mixed/other	1 (2)
Missing	2 (5)
**Hispanic/Latino**,** n (%)**	
Yes	1 (2)
No	36 (84)
Missing	6 (14)
**Years in practice**,** median (IQR)**	21.0 (13.5–28.0)

Implementation outcomes demonstrated excellent internal consistency across constructs (α = 0.96–0.99); we therefore report scale means here, with item-level distributions provided in Additional file 1. On a 5-point scale, providers rated the implementation strategy as highly acceptable (AIM mean = 4.33, SD = 0.82), reporting that the multicomponent strategy was appealing and that they welcomed its use. Appropriateness ratings were similarly strong (IAM mean = 4.37, SD = 0.78), reflecting providers’ perception of the strategy as a good fit for their practice. Sustainment was rated lower than acceptability and appropriateness (PRESS mean = 3.95, SD = 0.85). Most providers reported that the on-time HPV vaccination practices had been incorporated into routine practice. Item-level distributions for all three constructs are shown in Additional file 1.

### Implementation costs

The mean total implementation cost was $10,021 per practice (range: $6,494- $14,148) over the 24-month implementation period (Table 2). This estimate comprised practice facilitator implementation delivery costs and practice personnel costs. Practice facilitators accounted for 45.2% of total costs, while practice personnel time accounted for 54.8%.

**Table 2:** Mean hours of implementation activities and costs per practice over the 24-month implementation period

Implementation delivery	Mean hours per practice	Mean total compensation per practice^a^	% of total cost
**Practice facilitators (PF)** ^b^			
Quality improvement meeting	78.1	$3766	37.6%
Interview	10.0	$483	4.8%
Training/coaching	1.4	$68	0.7%
Data collection	3.5	$169	1.7%
PF subtotal	93.0	$4485	44.8%
PF transportation (mileage)^c^	-	$47	0.5%
**Total PF per practice**	**93.0**	**$4532**	**45.2%**
**Pediatric practice personnel**			
Primary care physicians	23.4	$3830	38.2%
Nurse practitioners	6.8	$598	6.0%
Registered nurses	2.0	$131	1.3%
Medical assistants and other staff	1.9	$55	0.5%
Health services managers	9.6	$876	8.7%
**Total practice personnel per practice**	**43.7**	**$5489**	**54.8%**
**Total cost per practice (PF + Practice personnel)**	**136.7**	**$10,021**	**100%**

a. Personnel costs calculated using 2023 U.S. Bureau of Labor Statistics national mean hourly wages, then adjusted to reflect total compensation by multiplying by 1.416. The sum of individual subcategories may differ slightly from the total values presented due to rounding. National mean hourly wages: practice facilitator, $34.04; primary care physician, $115.77; nurse practitioner, $61.78; registered nurse, $45.42; medical assistant and other, $20.84; health services manager, $64.64

b. PF activity types: quality improvement meeting, facilitator-led meetings and Plan-Do-Study-Act cycles; interview, provider interviews to assess practice needs and implementation progress; training/coaching, educational vaccine in-service for staff and providers; data collection, chart audits and data extraction for vaccination rate feedback

c. Valued at 2023 IRS standard mileage rate ($0.655/mile); not adjusted for benefits

From the implementer perspective, 2 practice facilitators logged an average of 93.0 h per practice over the 24-month implementation. This time was allocated primarily to quality improvement meetings (78.1 h, 84.0%), followed by provider interviews to assess needs (10.0 h, 10.8%), data collection (3.5 h, 3.8%), and educational vaccine in-service for staff and providers (1.4 h, 1.5%). These practice facilitators hours corresponded to a total cost of $4,485 per practice. After including mileage, the mean implementation delivery cost was $4,532 (range: $2,895-$5,943).

From the pediatric practice perspective, practice staff invested an average of 43.7 h per practice in implementation activities, with PCPs contributing the most (23.4 h, 53.5%), followed by practice managers (9.6 h, 21.9%), nurse practitioners (6.8 h, 15.6%), registered nurses (2.0 h, 4.6%), and medical assistants and other staff (1.9 h, 4.3%). The mean practice personnel cost was estimated at $5,489 per practice (range: $2,945-$9,643).

### Cost-effectiveness

Cost-effectiveness was assessed at two levels. At the practice level, based on a 6.2 percentage-point improvement in HPV vaccination initiation during the sustainment period, the estimated cost to achieve 1 percentage-point improvement was $1,616 per practice (Table 3). For series completion (8.6 percentage-point improvement), the estimated cost per 1 percentage-point improvement was $1,165 per practice.

**Table 3:** Cost-effectiveness estimates per percentage-point improvement and per additional adolescent vaccinated at 12 months post-implementation

Cost per percentage-point improvement (per practice)	HPV vaccination initiation^a^	HPV vaccination completion^a^
Practice facilitator	$731	$527
Pediatric practice personnel	$885	$638
**Total**	$1616	$1165
**Cost per additional adolescent vaccinated** ^**b**^		
Practice facilitator	$331	$238
Pediatric practice personnel	$400	$289
**Total**	$731	$527 ($276-$6475)^c^

a. Costs based on prior RCT estimates at sustainment period (12 months post-implementation). HPV vaccination initiation: 6.2 percentage points difference (95% CI: -0.4 to 12.8); HPV vaccination completion: 8.6 percentage points difference (95% CI: 0.7 to 16.4)

b. Estimated additional vaccinations were calculated as intervention group adolescents (*n* = 2,211) × percentage-point difference between groups at 12 months post-implementation. Both initiation and completion estimates reflect the same total implementation cost per practice ($10021). The lower cost per additional vaccination for completion reflects the larger effect magnitude compared to initiation

c. Range reflects cost per additional vaccination at the upper and lower bounds of the 95% CI for completion (16.4 and 0.7 percentage points, respectively), reflecting uncertainty in effect estimates rather than variation in implementation costs. HPV vaccination initiation 95% CI crosses zero; therefore, range is not reported

At the patient level, the estimated cost per additional adolescent vaccinated was $731 for HPV vaccination initiation (facilitator: $331, practice: $400) and $527 for series completion (facilitator: $238, practice: $289). These two estimates reflect the same total implementation cost ($10,021 per practice) applied to two different outcomes. The lower cost per adolescent for HPV vaccination series completion reflects the larger sustained effect on completion compared to initiation at sustainment follow-up.

## Discussion

This study advances the implementation science literature by explicitly linking provider-perceived implementation outcomes (acceptability, appropriateness, sustainment) with detailed, dual-perspective implementation costs within a pragmatic randomized trial. While prior studies have evaluated these domains separately, our findings provide empirical insight into how perceived strategy fit aligns with the magnitude and distribution of real-world resource investment required to achieve and sustain practice change.

Post-implementation surveys demonstrated providers viewed the multicomponent strategy as acceptable, appropriate, and well suited to their practice. These favorable ratings are consistent with the hypothesized IRLM pathways (Fig. 1), in which the strategy was designed to address practice-level barriers through mechanisms such as barrier resolution, increased performance awareness, and improved provider confidence. As these mechanisms were not directly measured, they should be interpreted as hypothesized pathways. The modest time required of practice personnel (44 h per practice, or 1.8 h per month), much of which was incorporated into existing meetings, likely minimized disruption and may have supported the high acceptability and appropriateness ratings. Notably, ratings remained high despite delivery during the COVID-19 pandemic, demonstrating the strategy’s capacity for contextual adaptation. Practice facilitators delivered tailored strategy components to each practice and shifted to virtual formats (e.g., Zoom-based facilitation), preserving engagement and reducing logistical burden in a way that may have enhanced implementation feasibility. This feasibility may in part reflect the increased flexibility associated with virtual engagement during the pandemic, which may differ from fully in-person implementation contexts.

Sustainment scores remained positive, although lower than acceptability and appropriateness ratings. Most providers reported that strategy components had been integrated into routine practice. The combination of practice facilitation with systematic quality improvement processes, tailored to each practice’s context, may explain this sustainment potential. This aligns with the Dynamic Sustainability Framework, which posits that sustained or even increasing benefit is achievable when strategies optimize fit with the practice context [26]. At the same time, sustainment observed following a period of pandemic-related disruption may reflect both adaptation to new workflows and recovery of preventive care activities, which should be considered when interpreting these findings.

This sustainment finding has direct implications for interpreting implementation costs. The mean cost of $10,021 per practice represents a time-limited investment in a multicomponent strategy designed for sustainment. This provides insight into the effort required to achieve favorable provider-perceived implementation outcomes and highlights a distinctive feature of the facilitation-based strategy: the external facilitator offloaded the implementation burden and provided support, particularly during the COVID-19 pandemic. External facilitation accounted for 45% of total implementation cost, corresponding to an average of 93 h of facilitator effort per practice. For practice personnel, the implementation strategy translated into an average of 44 h per practice, representing 55% of total implementation cost. Notably, cost also differed by perspective over time. Facilitation costs were non-recurring and ended when external support was withdrawn, whereas practices incurred some ongoing costs in maintaining activities internally, such as data review and reinforcement of workflow changes. For scale-up, this distinction is important because external facilitation requires initial implementation cost, while sustaining the gains requires modest continued effort from the practices.

Compared with other HPV vaccination strategies, our cost of delivering multicomponent strategy was higher. However, direct comparison of cost is challenging because studies differ in outcome measurement, settings, strategy intensity [14–17], as well as costing methods [11]. Our approach to capturing and separating cost by perspective aligns with recent implementation cost analyses in other clinical contexts [27, 28], though such costing of an HPV vaccination strategy from perspectives remains limited. Among existing HPV vaccination strategy estimates, reported costs for less intensive strategies range from $1,420 per practice annually for mailed parent reminders and clinician audit reports ($47 to $202 per additional adolescent vaccinated) [14], to $2,003 per practice for a 6-month clinician online communication training ($110 per averted missed opportunity at well-child visits) [15]. Our multicomponent strategy required a higher initial investment, an estimated cost of $527 per additional adolescent completing the HPV vaccination series (facilitator: $238, practice: $289), reflecting the intensity of the facilitation-based strategy delivered over 24 months.

Assessing the value for money of facilitation-based strategies is complicated by their integrated design. Lower-cost implementation strategies do not inherently represent good value if they fail to produce meaningful or enduring clinical change [27]. In our study, the higher cost reflected the intensity of practice facilitation, which served as the integrating mechanism through which the other components were delivered. This external facilitation support was especially important for practices lacking internal facilitation or quality-improvement infrastructure. Therefore, this implementation cost should not be interpreted as the cost of any single component, but rather as the effort required to coordinate, tailor, and embed multiple components into routine practice. Sustainment was provider-reported and therefore reflects perceived rather than objectively verified durability. However, the reported persistence of practice changes after active facilitation ended is consistent with evidence that tailored facilitation builds internal capacity, supporting durable organizational change within primary care [29, 30]. 

From a broader population health perspective, the personnel investment required is modest relative to the long-term cancer prevention benefits. In the US, approximately 39,300 HPV-attributable new cancer cases are diagnosed each year [31], meanwhile the burden of screening and treating HPV-related disease exceeds over $9 billion annually [32]. Modelling evidence shows that scaling evidence-based HPV vaccination interventions could avert approximately 3,000 to 14,000 future cancers nationally, yielding a favorable health return on investment [33]. Although our study did not directly model long-term cost-effectiveness or downstream cancer outcomes, these findings provide important inputs for future economic evaluations and policy decision-making. Given the high acceptability, appropriateness, and reported sustainment of the multicomponent strategy, it may contribute to long-term health benefits and cost savings through cancer prevention.

Our findings suggest that this multicomponent strategy is well suited for broad adoption by community practices and integration into state-level efforts to advance HPV vaccination. By estimating implementation costs from both the practice facilitator (implementer) and practice (recipient) perspectives, this study provides actionable information for resource planning, budgeting, and adaptation. Quantifying implementation costs allows organizations to separately plan for external facilitation infrastructure and anticipated practice-level effort, supporting more realistic implementation planning. Future implementations should consider hybrid delivery models that combine virtual and in-person facilitation to balance cost efficiency with relationship-building and practice engagement. Together, these findings support the scalability of facilitation-based strategies, particularly when resource requirements are explicitly quantified and aligned with sustained implementation benefits across diverse practice settings.

### Limitations

Several limitations should be considered. First, the 60% survey response rate and reliance on self-reported data may introduce nonresponse and social desirability bias. Providers who were more engaged with the strategy may have been more likely to respond, potentially inflating estimates of acceptability and sustainment. Second, we were unable to disaggregate costs by individual implementation strategy, as practice facilitators played a central role in organizing and delivering multiple components simultaneously. Additionally, implementation outcomes were assessed following strategy exposure and pooled across study groups with differing timelines, limiting causal inference and introducing potential recall bias. Due to the predominance of virtual facilitation during the pandemic, some cost components were likely underestimated, particularly transportation (mileage); implementation costs in non-pandemic settings may therefore be higher. In addition, the costs of practices maintaining HPV-related activities after the strategy concluded were not included. Moreover, the study was conducted primarily in suburban practices within an established practice-based research network, which may limit generalizability to rural or under-resourced settings. While the IRLM informed conceptualization and interpretation, the proposed mechanisms were not directly measured, limiting our ability to empirically test pathways linking strategies to outcomes. Finally, the study was conducted during the COVID-19 pandemic, which likely influenced both implementation processes (e.g., increased virtual facilitation) and external contextual factors (e.g., disruptions in preventive care and changing vaccine perceptions).

## Conclusions

This secondary analysis of a cluster RCT demonstrated that providers found the multicomponent strategy highly acceptable, appropriate, and sustained 12 months after active facilitation ended, with minimal disruption to clinical operations. These findings provide evidence that multicomponent, facilitation-based implementation strategies may achieve sustained integration into routine pediatric primary care, even within complex and evolving healthcare environments. By pairing favorable provider-reported implementation outcomes with detailed dual-perspective cost data, this study moves beyond effectiveness to provide actionable evidence for health systems, policymakers, and practice networks seeking to adopt and scale evidence-based HPV vaccination strategies. The combination of favorable provider ratings and transparent resource requirements position this approach as a pragmatic and scalable model for improving preventive care delivery in pediatric settings. Future work should evaluate adaptation and implementation across diverse practice contexts to further inform large-scale dissemination and long-term population health impact.

## Supplementary Information

Below is the link to the electronic supplementary material.

Supplementary Material 1

## Data Availability

The datasets used and/or analyzed during the current study are available from the corresponding author on reasonable request.

## Abbreviations

AIM: Acceptability of Intervention Measure
CDC: Centers for Disease Control and Prevention
CI: Confidence interval
ERIC: Expert Recommendations for Implementing Change
HPV: Human papillomavirus
IAM: Intervention Appropriateness Measure
IQR: Interquartile range
IRLM: Implementation Research Logic Model
IRS: Internal Revenue Service
PCP: Primary care physicians
PRESS: Provider REport of Sustainment Scale
QI: Quality improvement
RCT: Randomized controlled trial
SD: Standard deviation
REDCap: Research Electronic Data Capture
