# Supplementary material for: Acceptability, appropriateness, sustainment, and implementation cost of a multicomponent strategy to increase HPV vaccination in pediatric primary care: secondary results of a cluster-randomized trial
Source: Implement Sci Commun. 2026 Aug 6;7:222. doi: 10.1186/s43058-026-01068-w (PMC13737714; doi:10.1186/s43058-026-01068-w)
Supplement: Supplementary file 1 [file 43058_2026_1068_MOESM1_ESM.docx]

**Additional file 1. Post-implementation survey outcomes: provider ratings of acceptability, appropriateness, and sustainment**

Acceptability (A), appropriateness (B), and sustainment (C) from 43 providers. One provider did not respond to the “welcomed” item (n=42). The sum of individual response categories in the figure may differ from the combined aggregate proportions reported in the text due to rounding. The term “intervention” refers to the multicomponent implementation strategy and the HPV vaccination practices promoted through that strategy.
